# Probing the Antiplasmodial Properties of Plakortinic Acids C and D: An Uncommon Pair of Marine Peroxide-Polyketides Isolated from a Two-Sponge Association of *Plakortis symbiotica* and *Xetospongia deweerdtae* Collected near Puerto Rico

**DOI:** 10.3390/life14060684

**Published:** 2024-05-27

**Authors:** Luis A. Amador, Emilee E. Colón-Lorenzo, Abimael D. Rodríguez, Adelfa E. Serrano

**Affiliations:** 1 Molecular Sciences Research Center, University of Puerto Rico, 1390 Ponce de León Avenue, San Juan 00926, Puerto Rico; luisalberto.amador@upr.edu; 2 Department of Microbiology and Medical Zoology, University of Puerto Rico School of Medicine, San Juan 00921, Puerto Rico; emilee.colon@upr.edu

**Keywords:** plakortinic acids C and D, *Plasmodium berghei*, antiplasmodial activity, *P. berghei* in vitro drug luminescence assay, erythrocyte cell lysis assay, ADMET

## Abstract

Plakortinic acids C (**1**) and D (**2**), an unseparable pair of endoperoxide polyketides isolated and purified from the symbiotic association of Caribbean Sea sponges *Plakortis symbiotica-Xestospongia deweerdtae*, underwent in vitro evaluation for antiplasmodial activity against the malaria parasite *Plasmodium berghei* using a drug luminescence assay. Initial screening at 10 µM revealed 50% in vitro parasite growth inhibition. The title compounds displayed antiplasmodial activity with an EC_50_ of 5.3 µM toward *P. berghei* parasites. The lytic activity against erythrocytes was assessed through an erythrocyte cell lysis assay, which showed non-lytic activity at lower concentrations ranging from 1.95 to 3.91 µM. The antiplasmodial activity and the absence of hemolytic activity support the potential of plakortinic acids C (**1**) and D (**2**) as promising lead compounds. Moreover, drug-likeness (ADMET) properties assessed through the pkCSM server predicted high intestinal absorption, hepatic metabolism, and volume of distribution, indicating favorable pharmacokinetic profiles for oral administration. These findings suggest the potential suitability of these metabolites for further investigations of antiplasmodial activity in multiple parasitic stages in the mosquito and *Plasmodium falciparum.* Notably, this study represents the first report of a marine natural product exhibiting the unique 7,8-dioxatricyclo[4.2.2.0^2,5^]dec-9-ene motif being evaluated against malaria.

## 1. Introduction

Malaria, an ancient disease, remains a significant worldwide threat [1,2,3]. The World Health Organization’s 2022 report revealed alarming numbers—with nearly 249 million cases worldwide, resulting in almost 608 thousand malaria deaths [4].

Protozoan parasites such as *Plasmodium falciparum*, *Plasmodium vivax*, *Plasmodium ovale*, *Plasmodium knowlesi*, and *Plasmodium malariae* [5] are the causal agents of malaria. Among these, *P. falciparum* and *P. vivax* [6] are the primary parasites responsible for causing malaria. Malaria become more complex due to the intricate interactions among the parasite cycle, human hosts, and the vectors that help its transmission [7]. Additionally, novel, effective compounds with the potential for treatment are urgently needed since *Plasmodium* parasites have developed resistance to almost all antimalarials on the market [8,9,10,11].

Malaria transmission mainly occurs through the bites of female *Anopheles* mosquitoes, which are carriers for spreading parasites [12]. Infected individuals harbor the parasites within red blood cells, facilitating transmission through mosquito bites, blood transfusions, organ transplants, and mother-to-child transmission during childbirth [13]. Malaria symptoms mimic influenza-like chills, headaches, and muscle fatigue [13]. Moreover, malaria can lead to complicated side effects such as anemia and cerebral or respiratory distress [5].

An obstacle in malaria eradication is the parasite’s resistance to current drug treatments, with both the parasite and the vector being extraordinarily adaptable [14,15,16]. The treatment for malaria depends on factors such as the type of the infecting parasite, the geographical location of the acquired infection, and the patient’s condition [17,18,19]. Therefore, finding specific drugs to treat malaria in the long term is challenging [14,20,21].

**Figure 1:**
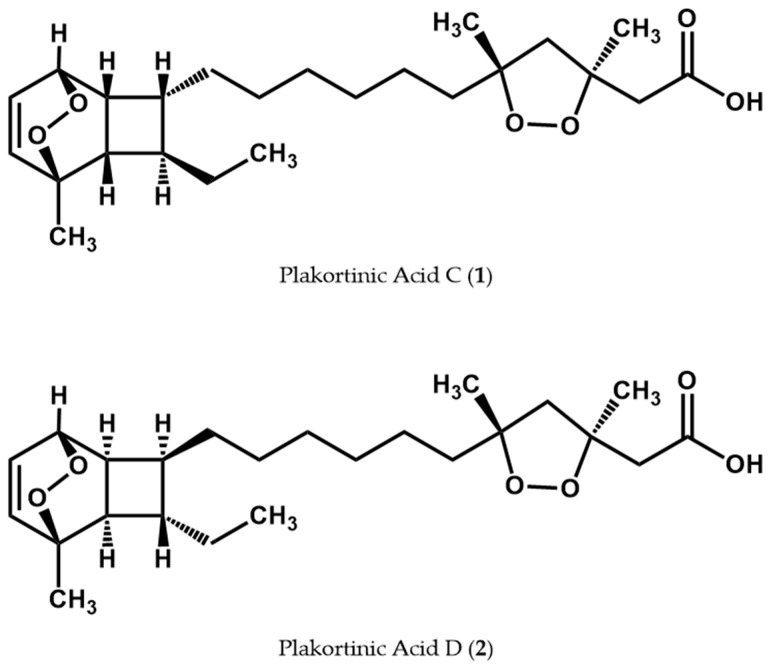
Chemical structures of plakortinic acid C (**1**) and plakortinic acid D (**2**).

Marine natural products have gained significance in developing drugs for treating malaria due to the diverse secondary metabolites produced by marine organisms. These compounds exhibit remarkable structural arrangements, resulting in various biological activities. Notably, natural products containing cyclic endoperoxides isolated from marine sponges belonging to the genera *Plakortis* and *Plakinastrella* have demonstrated significant antiplasmodial activity [22,23,24,25,26]. In pursuing novel therapeutic agents from marine sources, we recently disclosed two unprecedented natural products featuring two cyclic endoperoxide functionalities in their molecular structure. Figure 1 shows the chemical structures of these rare natural products identified as plakortinic acid C (**1**) and plakortinic acid D (**2**) [27].

These compounds were isolated together as an inseparable mixture from the symbiotic association of two Caribbean marine sponges, *Plakortis symbiotica*-*Xestospongia deweerdtae*, collected on Mona Island, off the west coast of Puerto Rico [27]. This rare sponge consortium, originally classified as an association between *Plakortis halichondrioides* and *Xetospongia deweerdtae* [28,29], has been shown to host molecules with unique chemical structures and exceptional bioactivities. For example, smenothiazole A, which displayed a MIC value of 4.1 μg/mL against *Mycobacterium tuberculosis* [30], along with mixtures of plakortinic acids A–D, some of which have demonstrated submicromolar activity against human A2058 melanoma and DU-145 prostate cancer cell lines [27,31], have been isolated from assortments of these two sponges. These findings underscore the potential of sponge symbiotic associations to generate structurally unique natural products with useful biological activities, making them attractive targets for future investigations in drug discovery.

This report presents the first antiplasmodial activity evaluation of a pair of compounds exhibiting the uncommon 7,8-dioxatricyclo[4.2.2.0^2,5^]dec-9-ene core. Herein, we screened the naturally occurring mixture of plakortinic acid C (**1**) and plakortinic acid D (**2**) [27] against the rodent malaria parasite, *Plasmodium berghei*, using an in vitro drug luminescence assay. Subsequently, we evaluated its toxicity through the erythrocyte cell lysis assay and the predicted absorption, distribution, metabolism, excretion, and toxicity (ADMET) properties. 

Our results showed that plakortinic acids C (**1**) and D (**2**) do not have toxicity against erythrocytes and exhibit drug-like properties supporting the potential to become an antimalarial drug candidate. Furthermore, these compounds represent a novel lead compound for further development as an antimalarial drug.

## 2. Materials and Methods

### 2.1. Sponge Material, Extraction, and Isolation

Fresh specimens of the sponge *Plakortis symbiotica* (Vicente, Zea & Hill, 2016) (phylum Porifera; class Homoscleromorpha; order Homosclerophorida; family Plakinidae) in association with *Xestospongia deweerdtae* (Lehnert & van Soest, 1999) (phylum Porifera; class Demospongiae; subclass Heteroscleromorpha; order Haplosclerida; family Petrosiidae) were collected by hand during scuba diving at depths from 90 to 100 ft off the coast of Mona Island, located on the western side of Puerto Rico (18°05.12′ N 67°53.22′ W), in July 2006. A voucher specimen (No. IM0609) is stored at the Chemistry Department of the University of Puerto Rico, Río Piedras Campus. 

### 2.2. General Experimental Procedures

Chemical reagents were procured from several suppliers, including Acros (Geel, Antwerpen, Belgium), Fluka (Charlotte, NC, USA), Sigma–Aldrich (Burlington, MA, USA), and TCI (Chuo-ku, Tokyo, Japan). Deuterated solvents were obtained from Sigma-Aldrich. Fourier transform infrared (FTIR) experiments were conducted using a Bruker Tensor 27 FTIR spectrometer. Nuclear magnetic resonance (NMR) data were acquired in CDCl_3_ with a Bruker DRX500 spectrometer. Chemical shifts were referenced to the corresponding solvent signals (δ_H_ 7.26 and δ_C_ 77.00 for deuterated chloroform). The spectra were processed using Mestrenova (Mnova 11.0 Mestrelab Research) software. The high-resolution electrospray ionization mass spectroscopy (HRESIMS) data were obtained at the Mass Spectrometry Laboratory of the School of Chemical Sciences, University of Illinois, using a VG 70-VSE (EI+, 70 eV). Optical rotations were measured in chloroform (CHCl_3_) with an Autopol IV automatic polarimeter using a 10 mm microcell. Thin layer chromatography (TLC) was carried out on precoated silica gel 60 GF254 or RP-18 silica gel 60 GF254 (Analtech, Newark, DE, USA). Gravity column chromatography was performed with normal and reversed-phase (RP-18) silica gel (35−75 mesh, Analtech). Visualization was achieved using UV light and/or an appropriate stain, such as iodine vapors on silica, sulfuric acid, or phosphomolybdic acid (PMA) ethanolic solution. Analytical reverse-phase high-performance liquid chromatography (RP-HPLC) was carried out using a Hypersil RP18 (250 × 4.6 mm i.d., 5 μm) column mounted to an Agilent 1260 series system controller equipped with a 1260 G1315D photodiode array detector and ChemStation software (B.04.02 SP2) from Agilent (Santa Clara, CA, USA), using HPLC grade solvents. 

### 2.3. Extraction and Isolation

The extraction and isolation methodologies were carried out as previously described [28]. The organisms were frozen and lyophilized before extraction. The dried specimens (395 g) were cut into small pieces and blended in a mixture of CHCl_3_-MeOH (1:1) (11 × 1 L). Following filtration, the crude extract was concentrated and stored under vacuum to yield a dark gum (100 g), which was suspended in H_2_O (2 L) and subjected to extraction with *n*-hexane (3 × 2 L), CHCl_3_ (3 × 2 L), and EtOAc (3 × 2 L). Concentration under reduced pressure yielded 16.4 g of the *n*-hexane extract as a dark brown oil, a portion of which (3.7 g) was chromatographed over Si gel (130 g) using mixtures of *n*-hexane-acetone of increasing polarity (0–100%). Based on TLC and ^1^H NMR analysis, a total of 11 fractions (A–K) were obtained. Purification of fraction B (659 mg) by silica gel (13.0 g) column chromatography using 100% CHCl_3_ as eluent afforded a mixture of plakortinic acids C (**1**) and D (**2**) (22 mg, 0.04% yield) as a 3:1 mixture of diastereomers. A small aliquot of the mixture of 1 and 2 treated with diazomethane followed by successive column chromatography over silica gel was suitable for structure elucidation work. 

### 2.4. Methylation of the Mixture of Plakortinic Acids C (**1**) and D (**2**)

A solution containing a mixture of **1** and **2** (10 mg, 0.024 mmol) in CHCl_3_ (8 mL) was added to a solution of diazomethane in ether (10 mL), and the resulting mixture was stirred at 25 °C for 2 h. The oily residue was chromatographed on a short silica gel plug (1.0 g), eluting with a mixture of *n*-hexane-acetone (95:5) to yield three fractions (I–III). Fraction II (15 mg) was submitted to reversed-phased HPLC chromatography (column: RP Analytical Hypersil 5µ C18, 250 × 4.6 mm) using isocratic elution with MeCN-H_2_O (85:15, flow rate: 0.80 mL/min). After HPLC purification, four subfractions were obtained (IIa–d). Subfraction IIc yielded plakortinic acid methyl esters C and D (6.0 mg, 53% yield) as an inseparable mixture of stereoisomers in a 3:1 ratio based on ^1^H NMR integration (see Figure S1 in the Supplementary Materials). This mixture was obtained as a colorless optically active oil: [α]^20^_D_ + 12.6 (c 0.54, CHCl_3_); IR (film) *v*_max_ 2932, 2854, 1738, 1456, 1376, 1210, 1088, 1012, 715 cm^−1^; HRESIMS analysis indicated a single [M+H]^+^ ion peak at *m*/*z* 437.2910 (calcd for 437.2903 [M+H]^+^), suggesting that each isomer possessed the same molecular formula of C_23_H_40_O_6_; plakortinic acid methyl ester C; ^1^H NMR (CDCl_3_, 500 MHz): δ 6.76 (dd, *J* = 8.1, 6.2 Hz, 1H), 6.34 (d, *J* = 8.1 Hz, 1H), 4.63 (t, *J* = 5.0 Hz, 1H), 3.69 (s, OCH_3_), 3.04 (ddd, *J* = 9.2, 9.0, 5.0 Hz, 1H), 2.78 (d, *J* = 14.5 Hz, 1H), 2.65 (d, *J* = 14.5, 1H), 2.47 (d, *J* = 12.6 Hz, 1H), 2.30 (dd, *J* = 8.3, 5.8, 1H), 2.22 (d, *J* = 12.6 Hz, 1H), 2.04 (m, 1H), 1.69 (m, 1H), 1.52 (m, 2H), 1.43 (s, 3H), 1.38 (m, 1H), 1.35 (m, 2H), 1.32 (m, 2H), 1.32 (s, 3H), 1.28 (m, 1H), 1.28 (s, 3H), 1.25 (m, 4H), 1.13 (m, 2H), 0.81 (t, *J* = 7.3, 3H); ^13^C NMR (CDCl_3_, 125 MHz): δ 171.1 (C), 135.8 (CH), 132.6 (CH), 86.4 (C), 83.9 (C), 77.3 (C), 72.5 (CH), 55.4 (CH_2_), 51.7 (OCH_3_), 44.8 (CH), 44.0 (CH_2_), 42.5 (CH), 41.3 (CH), 39.6 (CH_2_), 35.2 (CH), 30.7 (CH_2_), 29.9 (CH_2_), 29.7 (CH_2_), 29.6 (CH_2_), 28.3 (CH_2_), 24.4 (CH_2_), 24.1 (CH_3_), 23.2 (CH_3_), 20.5 (CH_3_), 11.9 (CH_3_); plakortinic acid methyl ester D; ^1^H NMR (CDCl_3_, 500 MHz): δ 6.59 (dd, *J* = 8.1, 6.2 Hz, 1H), 6.52 (d, *J* = 8.1 Hz, 1H), 4.61 (t, *J* = 5.0 Hz, 1H), 3.69 (s, OCH_3_), 3.04 (ddd, *J* = 9.2, 9.0, 5.0 Hz, 1H), 2.76 (d, *J* = 14.5 Hz, 1H), 2.63 (d, *J* = 14.5, 1H), 2.66 (dd, *J* = 8.3, 5.8, 1H), 2.54 (d, *J* = 12.6 Hz, 1H), 2.12 (d, *J* = 12.6 Hz, 1H), 2.00 (m, 1H), 1.69 (m, 1H), 1.52 (m, 2H), 1.45 (s, 3H), 1.38 (m, 1H), 1.35 (m, 2H), 1.35 (s, 3H), 1.32 (m, 2H), 1.28 (m, 1H), 1.28 (s, 3H), 1.25 (m, 4H), 1.13 (m, 2H), 0.76 (t, *J* = 7.4 Hz, 3H); ^13^C NMR (CDCl_3_, 125 MHz): δ 171.1 (C), 136.9 (CH), 131.4 (CH), 86.6 (C), 83.8 (C), 76.9 (C), 73.2 (CH), 55.4 (CH_2_), 51.6 (OCH_3_), 44.8 (CH), 44.1 (CH_2_), 42.0 (CH), 39.3 (CH), 38.8 (CH_2_), 38.2 (CH), 30.0 (CH_2_), 29.7 (CH_2_), 29.6 (CH_2_), 28.3 (CH_2_), 27.4 (CH_2_), 24.8 (CH_2_), 24.0 (CH_3_), 23.1 (CH_3_), 21.9 (CH_3_), 12.4 (CH_3_). The NMR spectra of compounds **1** and **2** and those of their respective methyl esters are included as Figures S1–S4 in the Supplementary Materials. 

### 2.5. Antiplasmodial Activity against the Parasite Plasmodium berghei

The antiplasmodial activity of the mixture of plakortinic acids C (**1**) and D (**2**) was evaluated using the *P. berghei* GFP-Luc_ama1_ (1037cl1) parasite line, which expresses the green fluorescent protein and the firefly luciferase genes under the control of the ama-1 schizont-specific promoter. The *P. berghei* GFP-Luc_ama1_ (1037cl1) parasite line was used to assess antiplasmodial activity and the half-maximal effective concentration (EC_50_) by in vitro drug luminescence assay, as previously described [32,33]. Chloroquine diphosphate salt (Sigma-Aldrich^®^—Cat. No. C6628) was used as a standard antimalarial drug in all experiments. Chloroquine diphosphate salt was used as a control (at a concentration of 100 nM) to assess complete inhibition of blood stage development. The mixture of compounds was dissolved in 100% DMSO to obtain a 10 mM stock solution, which was aliquoted and stored at −20 °C. Dilutions were prepared using RPMI 1640 medium supplemented with 20% heat-inactivated fetal bovine serum (FBS, Gibco^®^, Waltham, MA, USA) and 10,000 IU/mL of neomycin solution (Sigma-Aldrich^®^). Furthermore, these dilutions were prepared 24 h before the experiment and stored at 4 °C. Initial testing was performed at 10 µM in triplicate. Since *Plasmodium* growth inhibition was ≥50%, a dose–response curve analysis was conducted using eight different compound concentrations. Data analysis was conducted as previously described [32,33]. The EC_50_ was calculated using GraphPad Prism 6 software. Dose–response curves were produced in three independent experiments, each in triplicate. 

### 2.6. Erythrocyte Cell Lysis Assay

The mixture of plakortinic acid C (**1**) and D (**2**) was tested at ten serial dilutions against fresh mouse erythrocyte at 1% hematocrit in Dulbecco’s phosphate buffered saline (DBPS, Gibco^®^) in V-bottom microplates (Corning^®^ 96 well TC-treated microplate). The microplates were incubated for 24 h at 37 °C. Following incubation, the microplates were centrifuged for 5 min at 2000 rpm, and 50 µL of the supernatant was transferred to a new flat-bottom microplate (BD Falcon^®^). The QuantiChrom^TM^ Hemoglobin Assay Kit (BioAssay Systems (Hayward, CA, USA), Cat. No. DIHB-250) was used per the manufacturer’s instruction to determine the amount of hemoglobin released into the supernatant, as described previously [34]. Blood (1% hematocrit) with DBPS was used as a negative control for no cell lysis, and DBPS was used as a blank. Molecular biology grade saponin was purchased from Sigma-Aldrich^®^ (Cat. No. 47036). Saponin at 100 µg/mL was used as a positive control for 100% cell lysis. The mixture was tested in three independent experiments, each in triplicate. Calculations and data analysis were performed using Excel (Version 2404 Build 16.0.17531.20152 64-bit), and graphs were generated using GraphPad Prism 6 software [33].

### 2.7. In Silico Predicted ADMET Properties

As previously reported, the pkCSM server, a free web interface (http://structure.bioc.cam.ac.uk/pkcsm, accessed on 25 February 2024), was used to define the predicted absorption, distribution, metabolism, excretion, and toxicity (ADMET) parameters [35]. The pkCSM used the simplified molecular input line entry system (SMILES) string for prediction. ChemDraw Prime (version 20.0.0.41) was used to draw the chemical structures of compounds **1** and **2**, which were then saved as MDL MOL files and used to generate SMILES names using the Online SMILES Translator and Structure File Generator of the National Cancer Institute (https://cactus.nci.nih.gov/translate/, accessed on 19 February 2024). The predicted ADMET results were analyzed as recommended by Pires et al. [35].

## 3. Results

### 3.1. In Vitro Drug Luminescence Assay against Plasmodium berghei

**Figure 2:**
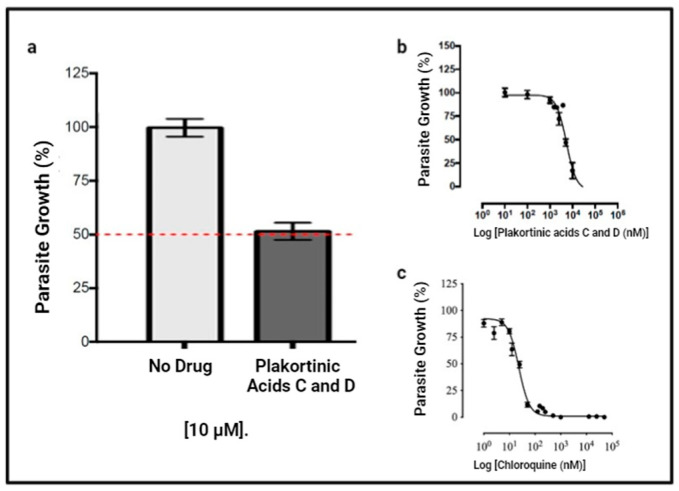
Antiplasmodial activity of the mixture of plakortinic acid C (**1**) and D (**2**). (**a**) Initial antiplasmodial activity at 10 µM. The red dashed line indicates 50% parasite growth. The mixture inhibited approximately 50% of parasite growth at 10 µM. The data represent one biological experiment in triplicate, and the bars represent the standard deviation. (**b**) Dose–response curve of plakortinic acids C (**1**) and D (**2**). The mixture showed *P. berghei* growth inhibition with an EC_50_ = 5318 nM (95% CI 4292–6589 nM). The data are the means ± SEM and represent three independent experiments, each in triplicate. (**c**) Dose–response curve of chloroquine as the positive control. The data are the means ± SEM and represent three independent experiments, each in triplicate. Chloroquine displayed *P. berghei* growth inhibition with an EC_50_ = 23.23 nM (95% CI = 20.72–26.04 nM).

The antiplasmodial activity of the mixture of plakortinic acid C (**1**) and D (**2**) was examined using the *P. berghei* in vitro drug luminescence assay [32,33]. Initial screening was performed at 10 µM. The compounds displayed 50% parasite growth inhibition at 10 µM (Figure 2a) compared to the control group (100%), indicating antiplasmodial activity. Subsequently, the mixture was subjected to a dose–response curve analysis, which showed the inhibition of *P. berghei* intra-erythrocytic growth with an EC_50_ of 5318 nM (5.3 µM) (Figure 2b). Chloroquine was used as a positive control, showing an EC_50_ of 23.23 nM (Figure 2c). 

### 3.2. Erythrocyte Cell Lysis Assay

**Figure 3:**
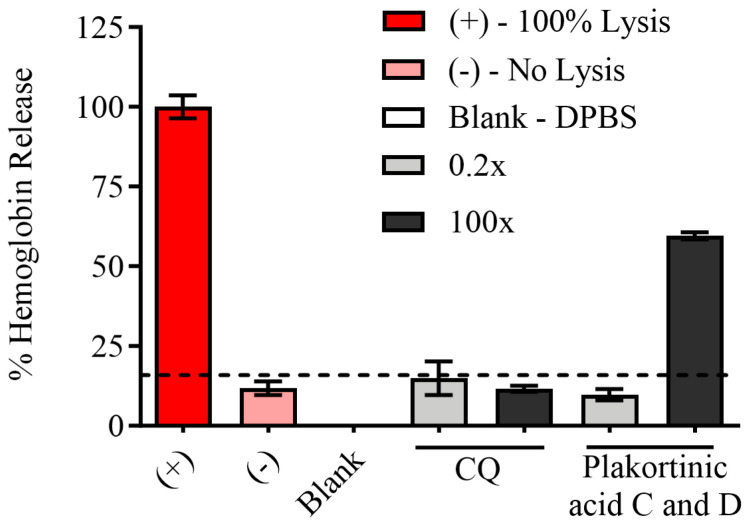
An erythrocyte cell lysis assay was used to evaluate the hemolytic effect of the mixture of plakortinic acids C (**1**) and D (**2**). The hemolytic effect of the mix on erythrocytes was assessed by measuring the release of hemoglobin in the supernatant. Experimental controls include 100 mg/mL saponin for 100% cell lysis as a positive control, blood with Dulbecco’s phosphate buffered saline DPBS (1% hematocrit) for no cell lysis as a negative control, and DPBS as a blank. The mixture of compounds was tested at ten dilutions ranging from 0.2-fold to 100-fold of its EC_50_ value from the antiplasmodial dose–response curve. The graph depicts the compounds’ concentrations at 0.2-fold and 100-fold. The data represent one biological experiment in triplicate, and the bars are the standard deviation. Preliminary results demonstrate no lytic activity of plakortinic acid C (**1**) and D (**2**) against erythrocytes at low concentrations (1.95–3.91 µM).

An erythrocyte cell lysis assay was performed to assess the potential toxicity of the mixture of plakortinic acids to erythrocytes by evaluating hemoglobin release. The erythrocyte cell lysis was evaluated at ten serial dilutions ranging from 0.2-foldto 100-fold above its EC_50_ against parasites. The compounds did not induce hemolysis of erythrocytes at low concentrations of 1.95–3.91 µM (Figure 3), indicating that the antiplasmodial effect of plakortinic acid C (**1**) and D (**2**) at these lower concentrations is not attributed to toxicity against erythrocytes. 

### 3.3. In Silico Predicted ADMET Properties of the Mixture of Plakortinic Acid C (**1**) and D (**2**)

Evaluation of drug-likeness is an important step for drug discovery and development. To evaluate the drug-likeness of the mixture of plakortinic acid C (**1**) and D (**2**), predicted ADMET parameters were determined using the pkCSM server [35]. The predicted ADMET of the plakortinic acids was compared to the antimalarial drug Chloroquine (Table 1).

There are seven predictors for absorption, including water solubility, membrane permeability in the colon cancer cell line (Caco2), intestinal absorption, skin permeability levels, and P-glycoprotein substrate or inhibitor (Pgp subs, Pgp I/II inh). The mixture of plakortinic acids was predicted to be water-soluble with a value of −4.392 log mol/L, similar to the predicted values of chloroquine CQ (−4.249 log mol/L). Caco2 permeability is considered high when the predicted value is >0.90. The mixture of compounds had a predicted value of 0.635 log Papp, indicating less Caco2 permeability than CQ (1.624 log Papp). The mixture was predicted to have high intestinal absorption with an estimated value of 93.557%, indicating a higher intestinal absorption than CQ (89.95%). Skin permeability is a parameter for transdermal drug delivery. Similarly to CQ, the compounds’ mixture was predicted to be skin permeable with an estimated value of −2.679 log Kp and −2.735 log Kp, respectively. While the mix of **1** and **2** was not predicted to be a P-glycoprotein (PgP) substrate nor a PgP I inhibitor, it was, however, predicted to be a PgP II inhibitor.

For distribution, there are four predictors, including volume of distribution (VDss), fraction unbound, blood–brain barrier (BBB) permeability, and central nervous system (CNS) permeability. The VDss is a parameter that describes the theoretical volume in which a drug needs to be evenly distributed to produce the same plasma concentration. The mixture was predicted to have a VDss of −0.121 log L/kg. In comparison, CQ had a VDss of 1.332 log L/kg, indicating a notably lower estimated volume of distribution for **1** and **2** compared to CQ. The mixture of compounds was predicted to have unbound fraction values of 0.089 Fu, similar to CQ (0.191 Fu). BBB and CNS permeability are parameters to estimate drug distribution in the brain. According to BBB permeability, compounds with log BB > 0.3 are suggested to cross the BBB readily, while compounds with log BB < −1 cross poorly. The plakortinic acids mixture had a predicted value of −0.681 log BB, and therefore was predicted to be poorly distributed to the brain, while CQ had a predicted value of 0.349 log BB, and therefore was predicted to cross the BBB. According to CNS permeability, compounds with a log PS > −2 are suggested to penetrate the CNS, while those with log PS < −3 cannot penetrate the CNS. The compounds’ mixture was predicted to penetrate the CNS with a permeability value of −2.894 log PS, similar to CQ (−2.191 log PS).

For metabolism, there are seven predictors based on the CYP models for substrates or inhibition. The mixture of plakortinic acid C (**1**) and D (**2**) is not a CYP substrate or inhibitor, except for the CYP3A4 substrate (Table 1). There are two predictors for excretion: the total clearance and the renal OCT2 substrate. The renal OCT2 substrate predictor evaluates a drug’s potential for kidney secretion, while total clearance measures the overall removal of a drug from the body via both renal and hepatic pathways. The mixture of acids was predicted to have total clearance with an estimated value of 0.953 log mL/min/kg, similar to CQ (1.092 log mL/min/kg). Thus, the mixture of carboxylic acids **1** and **2** is not a substrate of the OCT2 pathway.

The parameters that predict toxicity include the AMES test (carcinogenicity), hERG inhibition I/II (cardiotoxicity), hepatotoxicity, and skin sensitization. Based on the AMES toxicity prediction, the plakortinic acid mixture is not mutagenic and, according to the prediction, is not an hERG I/II inhibitor. Based on the projections, these natural product mixtures do not cause hepatotoxicity or skin sensitization.

Overall, the mixture of plakortinic acid C (**1**) and D (**2**) displayed predicted ADMET properties similar to CQ, suggesting a favorable compound disposition. Based on ADMET predictions, this blend of naturally occurring carboxylic acids is a promising lead compound that should be further developed.

## 4. Discussion

The oceans harbor a rich reservoir of bioactive compounds with potential therapeutic properties against various diseases, including malaria [36,37]. Marine-derived compounds exhibit antimalarial activity through different mechanisms, such as disrupting parasite metabolism or interfering with essential cellular processes [38,39,40,41,42]. Their exploration offers hope for overcoming drug resistance, a pressing challenge in malaria treatment. Thus, harnessing marine natural products for drug discovery presents a valuable strategy in combating infectious diseases such as malaria.

Malaria represents a major global health challenge due to the rise of multidrug resistance. The discovery of novel antimalarial drugs is crucial for treating and controlling the disease, which ultimately helps with malaria eradication. Chloroquine, a commonly used drug in the treatment of malaria, has faced prevalent resistance in malaria-endemic regions, diminishing its effectiveness in combating the disease [43,44,45,46,47,48,49,50]. Additionally, its use has been associated with various adverse effects, ranging from gastrointestinal disturbances to more severe conditions such as cardiotoxicity and retinopathy [51,52,53,54]. The persistent parasite resistance and the adverse effects associated with chloroquine, compounded by the emergence of increasing parasite resistance to artemisinin [55,56,57,58,59,60], a drug approved in the 1990s and utilized in combination therapies to combat malaria infections, particularly those caused by chloroquine-resistant parasite strains [61,62], highlight the pressing demand for alternative antimalarial drugs with enhanced efficacy, safety profiles, and mechanisms of action. Moreover, discovering novel antimalarial agents, whether sourced synthetically or from nature, is imperative for effective malaria control and reducing the global burden of this deadly disease. In this context, evaluating a mixture of plakortinic acids C (**1**) and D (**2**) as a potential antiplasmodial agent via a *P. berghei* in vitro drug luminescence assay provides valuable insights into its potential therapeutic efficacy. Indeed, the utilization of the *P. berghei* model in such investigations is well-established [63,64,65], serving as a critical preclinical phase in drug development due to its close resemblance to human malaria parasites in terms of life cycles and pathophysiology [66]. Additionally, the feasibility of managing *P. berghei’s* complete life cycle under laboratory conditions enhances its utility as a relevant model organism [67]. The findings indicate that the title compounds demonstrate significant antiplasmodial activity against *P. berghei* under in vitro conditions with an EC_50_ of 5.3 µM. This demonstrated equipotency with the antiplasmodial EC_50_ values reported for market drugs such as azithromycin, doxycycline, or proguanil, which are used in the treatment of multidrug-resistant P. *falciparum* malaria (Table 2) [68,69,70]; thereby positioning plakortinic acids C (**1**) and D (**2**) as potential alternatives for commercial drugs for malaria treatment. Furthermore, analysis of their hemolytic activity reveals non-toxicity to erythrocytes at lower concentrations ranging from 1.95 to 3.91 µM, suggesting their suitability for further investigations in *P. falciparum* studies.

The prediction of ADMET properties is crucial in the initial phases of drug discovery and development. ADMET predictions streamline hit prioritization and lead optimization, improving the drug development process. The mixture of plakortinic acids was predicted to meet the absorption criteria, including high intestinal absorption. The predicted P-glycoprotein modulation suggests that the mixture of compounds may have favorable and less metabolism-based drug interactions. The drug distribution predictor, CNS permeability, indicates that **1** and **2** can penetrate the CNS. Drug excretion prediction suggests that the plakortinic acids are not substrates of the OCT2 pathway. The effectiveness of lead compounds relies on their toxicity. Predictions indicate that plakortinic acids C (**1**) and D (**2**) are not mutagenic, nor do they exhibit hepatotoxicity.

The ensuing mixture of plakortinic acids displays drug-likeness properties with favorable pharmacokinetic profiles for oral administration due to their predictions of high intestinal absorption, hepatic metabolism, and volume of distribution. Their ADMET profile closely resembles CQ, supporting their potential for further development as an antimalarial drug. Results of the ADMET predictions and the erythrocyte lysis assay support further studies on developing the mixture of carboxylic acids as an antimalarial drug.

Likewise, the observed antiplasmodial activity demonstrated by these compounds can be attributed to their unique chemical structure, which includes two endoperoxide ring systems (see Figure 1). Such structural features have consistently been highlighted for their fundamental role in conferring antiplasmodial activity [26,71,72]. This aspect adds significant value to the ongoing investigation of plakortinic acids C (**1**) and D (**2**), not only due to their uncommon and extraordinary molecular structures, but also due to the mixture’s notable efficacy as an antiplasmodial agent, as supported by the results presented here. Importantly, this study marks the first report of marine natural products exhibiting the unique 7,8-dioxatricyclo[4.2.2.0^2,5^]dec-9-ene motif being evaluated against malaria, underscoring the novelty and potential significance of the title compounds in malaria research. Consequently, further structure–activity relationship assays and rodent in vivo studies will be required to determine the suitability of these natural products for additional development. 

## 5. Conclusions

Our current data represent a noteworthy progression towards the procurement of novel antimalarial drugs from the sea. Plakortinic acid C (**1**) and plakortinic acid D (**2**) have shown outstanding antiplasmodial action, making them emerge as attractive lead compounds. Still, these compounds are only at the beginning of the long road to clinical application. More research is needed to clarify the complex relationship between their structure and action, improve their potency, comprehend their pharmacokinetic profile, and evaluate their toxicity. This is underscored by the fact that previous evaluation of the methyl ester mixture of **1** and **2** against a panel of 60 human cancer cells showed slight cytotoxicity at a concentration of 10 µM [27].

As we advance, our research will involve thorough in vivo testing to confirm compounds **1** and **2**’s medicinal potential as effective malaria fighters. If these efforts are successful, new antimalarial medications could be introduced, which would help address the urgent need for alternative therapies due to the emergence of drug resistance. Furthermore, investigating chemicals obtained from marine sources emphasizes the enormous potential of natural resources in drug development and stresses the significance of multidisciplinary cooperation in furthering global health initiatives. Our ultimate goals are to strengthen global healthcare and aid in the ongoing fight against malaria.

## Figures and Tables

**Table 1:** Predicted pharmacokinetic and toxicity properties of plakortinic acids C (**1**) and D (**2**). The predicted ADMET properties were generated using the pkCSM server. The antimalarial drug chloroquine (CQ) was used as a control.

Parameters	Predictors	CQ	Plakortinic Acids C (1) and D (2)	Unit
Absorption	Water solubility	−4.249	−4.392	log mol/L
	Caco2	1.624	0.635	log Papp
	Intestinal abs	89.95	93.557	% Absorbed
	Skin perm	−2.679	−2.735	log Kp
	Pgp subs	Yes	No	Yes/No
	Pgp I inh	No	No	Yes/No
	Pgp II inh	No	Yes	Yes/No
Distribution	VDss	1.332	−0.121	log L/kg
	Fraction unbound	0.191	0.089	Fu
	BBB perm	0.349	−0.681	log BB
	CNS perm	−2.191	−2.894	log PS
Metabolism	CYP2D6 subs	Yes	No	Yes/No
	CYP3A4 subs	Yes	Yes	Yes/No
	CYP1A2 inh	No	No	Yes/No
	CYP2C19 inh	No	No	Yes/No
	CYP2C9 inh	No	No	Yes/No
	CYP2D6 inh	Yes	No	Yes/No
	CYP3A4 inh	No	No	Yes/No
Excretion	Total clearance	1.092	0.953	log mL/min/kg
	Renal OCT2 subs	Yes	No	Yes/No
Toxicity	AMES	Yes	No	Yes/No
	Max tol dose	−0.167	−0.336	log mg/kg/day
	hERG I inh	No	No	Yes/No
	hERG II inh	Yes	No	Yes/No
	Oral rat LD50	2.85	2.128	mol/kg
	Oral rat LOAEL	1.026	0.526	log mg/kg_bw/day
	Hepatotoxicity	Yes	No	Yes/No
	Skin sens	No	No	Yes/No
	*T. pyriformis*	1.558	0.307	Numeric (log ug/L)
	Minnow	0.747	−1.011	Numeric (log mM)

**Table 2:** Comparison of antiplasmodial EC_50_ values for the mixture of plakortinic acid C (**1**) and plakortinic acid D (**2**) with selected market drugs.

Antiplasmodial Compound	EC_50_ Value (µM)
Plakortinic Acids (**1** and **2**)	5.3
Azithromycin	29.3 [68]
Doxycycline	10.1 [69]
Proguanil	7.4 [70]

## Data Availability

The data presented in this study are available in this article.

## Supplementary Materials

The following Supplementary Materials can be downloaded at: https://www.mdpi.com/article/10.3390/life14060684/s1, Figure S1: ^1^H-NMR spectrum (CDCl_3_, 700 MHz) of plakortinic acids C (**1**) and D (**2**); Figure S2: ^13^C-NMR spectrum (CDCl_3_, 175 MHz) of plakortinic acids C (**1**) and D (**2**); Figure S3: ^1^H-NMR spectrum (CDCl_3_, 500 MHz) of plakortinic acids C and D methyl esters and Figure S4: ^13^C-NMR spectrum (CDCl_3_, 125 MHz) of plakortinic acids C and D methyl esters.
