# Supplementary material for: Probing the Antiplasmodial Properties of Plakortinic Acids C and D: An Uncommon Pair of Marine Peroxide-Polyketides Isolated from a Two-Sponge Association of Plakortis symbiotica and Xetospongia deweerdtae Collected near Puerto Rico
Source: Life (Basel). 2024 May 27;14(6):684. doi: 10.3390/life14060684 (PMC11204963; doi:10.3390/life14060684)
Supplement: Supplementary file 1 [file life-14-00684-s001.zip › life-2986382-supplementary.pdf]

Probing the Antiplasmodial Properties of Plakortinic Acids C and D: An Uncommon Pair of Marine Peroxide-Polyketides Isolated from a Two-Sponge Association of *Plakortis symbiotica* and *Xetospongia deweerdtae* Collected near Puerto Rico

Luis A. Amador [1,‡], Emilee E. Colón-Lorenzo [2,‡], Abimael D. Rodríguez [1*] and Adelfa E. Serrano [2,*]

[1] Molecular Sciences Research Center, University of Puerto Rico, 1390 Ponce de León Avenue, San Juan, Puerto Rico 00926; luisalberto.amador@upr.edu (L.A.A)
[2] Department of Microbiology and Medical Zoology, University of Puerto Rico School of Medicine, San Juan, Puerto Rico 00921; emilee.colon@upr.edu (E.E.C.-L)
‡ These authors contributed equally
* Corresponding authors. adelfa.serrano@upr.edu (A.E.S.); abimael.rodriguez1@upr.edu (A.D.R.)

Supplementary Material

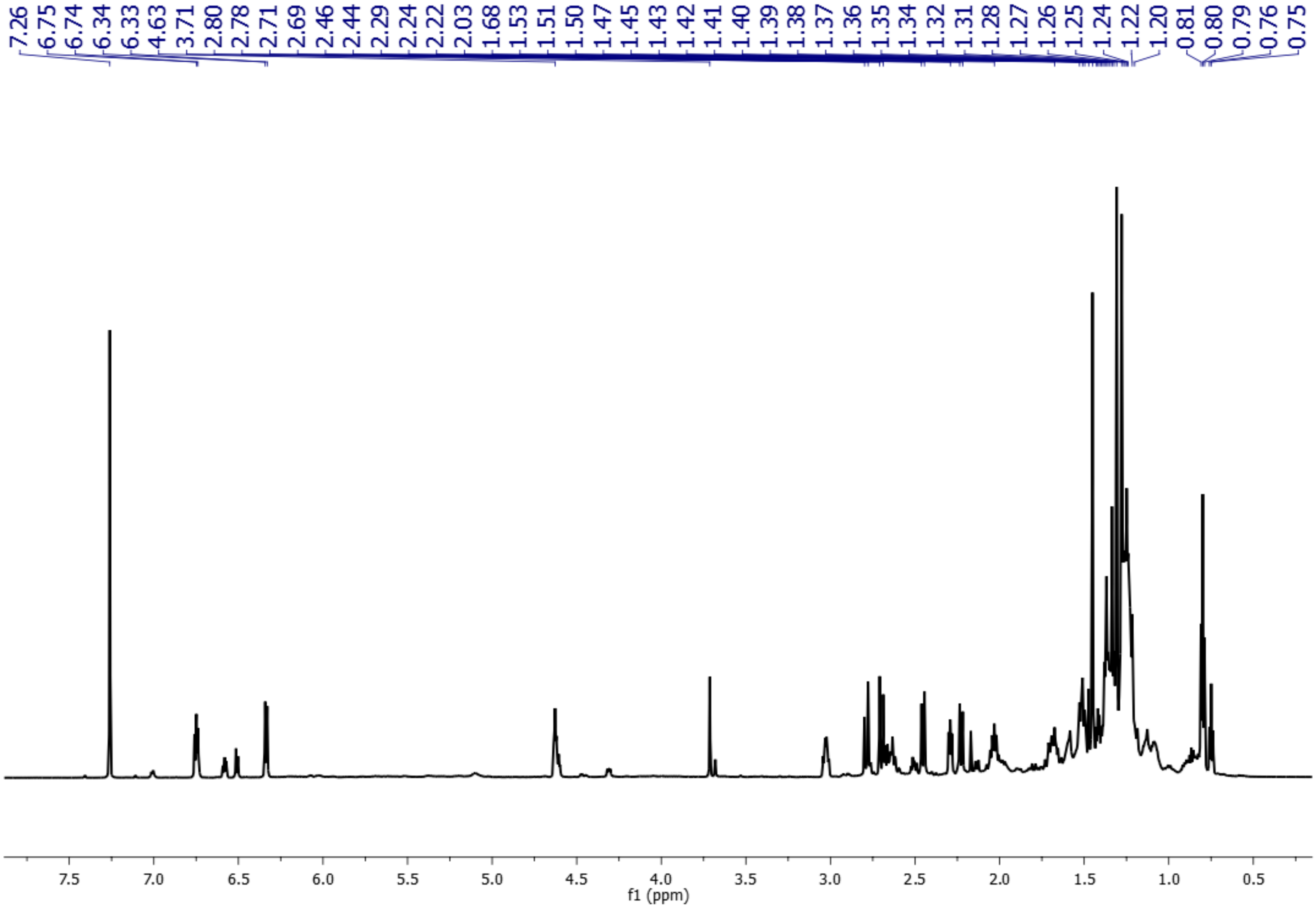

Figure S1. ^{1}H-NMR spectrum ($CDCl_3$, 700 MHz) of plakortinic acids C (**1**) and D (**2**).

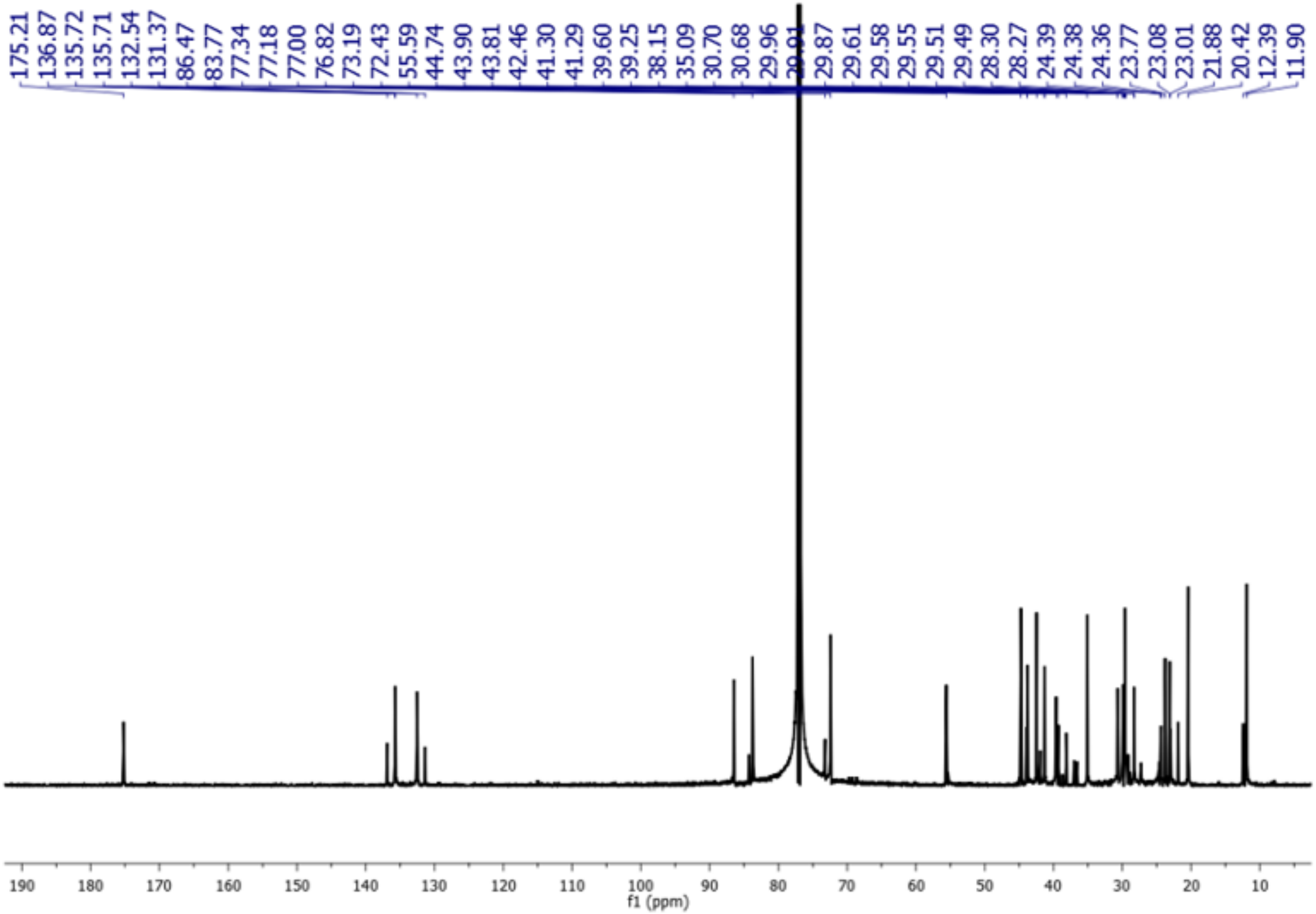

Figure S2. ^{13}C-NMR spectrum ($CDCl_3$, 175 MHz) of plakortinic acids C (**1**) and D (**2**).

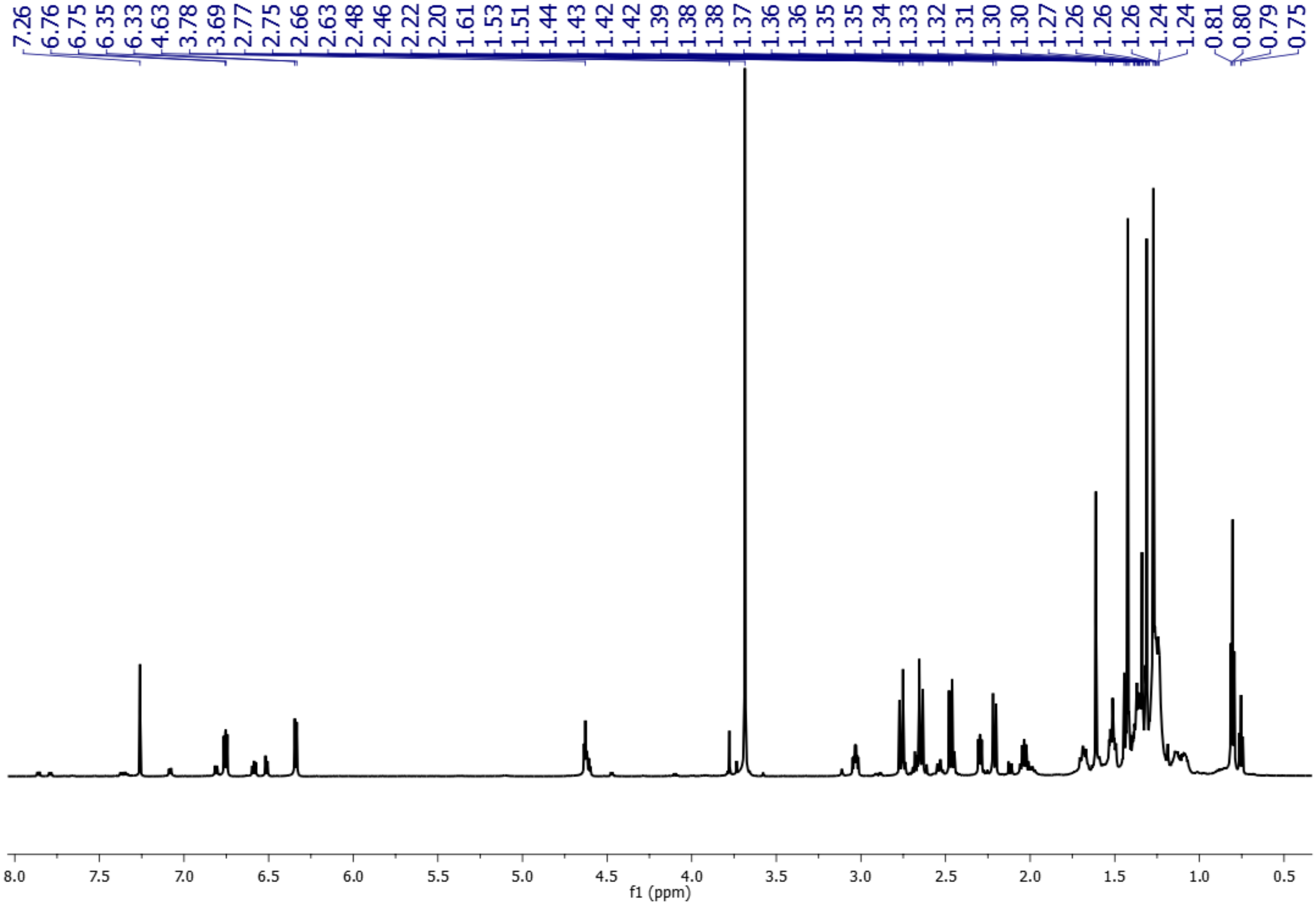

Figure S3. ^{1}H-NMR spectrum ($CDCl_3$, 500 MHz) of plakortinic acids C and D methyl esters.

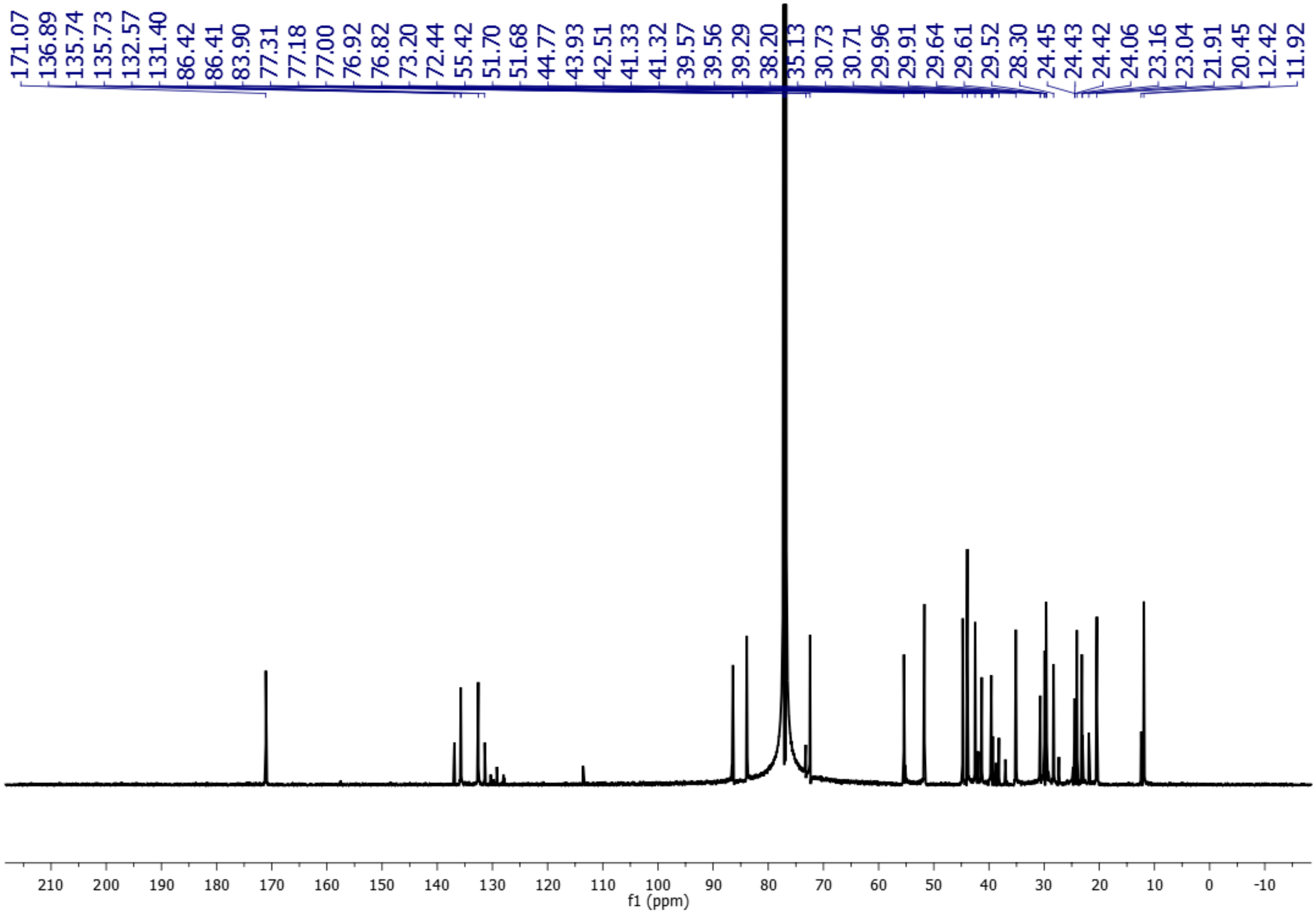

Figure S4. ^{13}C-NMR spectrum ($CDCl_3$, 125 MHz) of plakortinic acids C and D methyl esters.
